# ﻿On the identity and typification of *Violatenuis* Bentham (Violaceae)

**DOI:** 10.3897/phytokeys.252.138994

**Published:** 2025-02-20

**Authors:** Yan-Shuang Huang, Stephan W. Gale, Jinlong Zhang, Qiang Fan

**Affiliations:** 1 State Key Laboratory of Biocontrol and Guangdong Provincial Key Laboratory of Plant Stress Biology, School of Life Sciences, Sun Yat-sen University, Guangzhou, China Sun Yat-sen University Guangzhou China; 2 Flora Conservation Department, Kadoorie Farm and Botanic Garden, Tai Po, New Territories, Hong Kong SAR, China Flora Conservation Department, Kadoorie Farm and Botanic Garden Tai Po China

**Keywords:** Conservation, nomenclature, typification, *Viola* sect. *Plagiostigma* subsect. *Diffusae*

## Abstract

*Violatenuis* Benth. was first described by George Bentham in 1842, without any type specimen designated, but with a collection record by Richard Brinsley Hinds in 1841. Currently, *Violatenuis* is considered a synonym of another species according to Plants of the World Online (POWO). However, based on a combination of morphological and molecular evidence, we propose reinstating *Violatenuis* as a distinct species. Our analysis places it within Violasect.Plagiostigmasubsect.Diffusae, where it shows morphological similarities to five other species endemic to Guangdong Province, China. We designated the type of *V.tenuis* here and assigned additional KFBG specimens to the species.

## ﻿Introduction

Violasect.Plagiostigmasubsect.Diffusae, which is mainly distributed in Southeast Asia, has undergone a long and controversial history in classification. Wilhelm Becker first published this subsection as an unranked group *Diffusae* (Becker 1923) and it was then reclassified as V.sect.Nominiumser.Diffusae (Van Steenis 1934). Thereafter, Wang (1991) elevated the rank of this taxon as V.sect.Diffusae (W.Becker) C.J.Wang in *Flora Reipublicae Popularis Sinicae*. Recently, Marcussen et al. (2022) placed it in V.sect.Plagiostigma as subsect. Diffusae (W.Becker) C.C.Chang. The diversity of *Diffusae* species in China has long been underestimated, as 19 species have been discovered recently, 10 of which are new to science (Huang 2023). Amongst them, there is an unresolved name, *V.tenuis* Benth., published by George Bentham in 1842, whose identity we resolved here.

*Violatenuis*, a species having typical characters of V.subsect.Diffusae, with long peduncles, ovate-oblong leaf blades, thin rhizomes and flowers with short spurs and marginated style, was first collected in Hong Kong by Mr. Hinds and described by George Bentham in 1842 (Bentham 1842). Wilhelm Becker (1907) described a later homonym, *V.tenuis* W.Becker, from Brazil and reclassified the Hong Kong species as V.diffusasubsp.tenuis (Benth.) W.Becker (Becker 1921). The name *V.tenuis* Benth. was also accepted as a valid species in *Taiwania* (Wang and Huang 1990), but treated as a synonym of *V.diffusa* Ging. in “Flora Reipublicae Popularis Sinicae” (Wang 1991), “Flora of China” (Chen et al. 2007) and Plants of the World Online (Govaerts 2023).

Based on field surveys, molecular and morphological analyses, we provide evidence supporting the recognition of *V.tenuis* Benth. as a distinct species and its name should not be treated as a synonym. In this paper, we present updated descriptions and illustrations for the species.

## ﻿Materials and methods

We conducted field investigations and observations in Guangdong and Hong Kong during the flowering and fruiting periods of *Violatenuis*. Leaf material of the species and its relatives were collected and stored in zip-lock plastic bags with silica gel for comparisons and taxonomical treatment. We used a micrometer and a stereomicroscope to observe and measure morphological features to compile detailed descriptions, based on both fresh and dry specimens. Voucher specimens were deposited in the Herbarium of Sun Yat-sen University (SYS) and the Herbarium of Kadoorie Farm and Botanic Garden (KFBG).

Total DNA was extracted by using the modified CTAB method (Doyle and Doyle 1987). Previously reported primers ITS1 and ITS4 (White et al. 1990) were used to amplify the regions of partial internal transcribed spacer 1, 5.8S ribosomal RNA gene and partial internal transcribed spacer 2. PCR amplifications were performed following Fan et al. (2015). We downloaded the sequences of the species and related ones from NCBI and aligned them using MEGA 6.0 (Tamura et al. 2013) with ClusterW, then manually adjusted the alignment. Phylogenetic inferences were carried out with Maximum Likelihood (ML) using IQ-TREE 2.0.3 (Minh 2020). SYM+I+I+R2 was selected as the best–fit model and 2, 000 bootstraps were conducted to evaluate the confidence.

## ﻿Results

**Figure 1.**
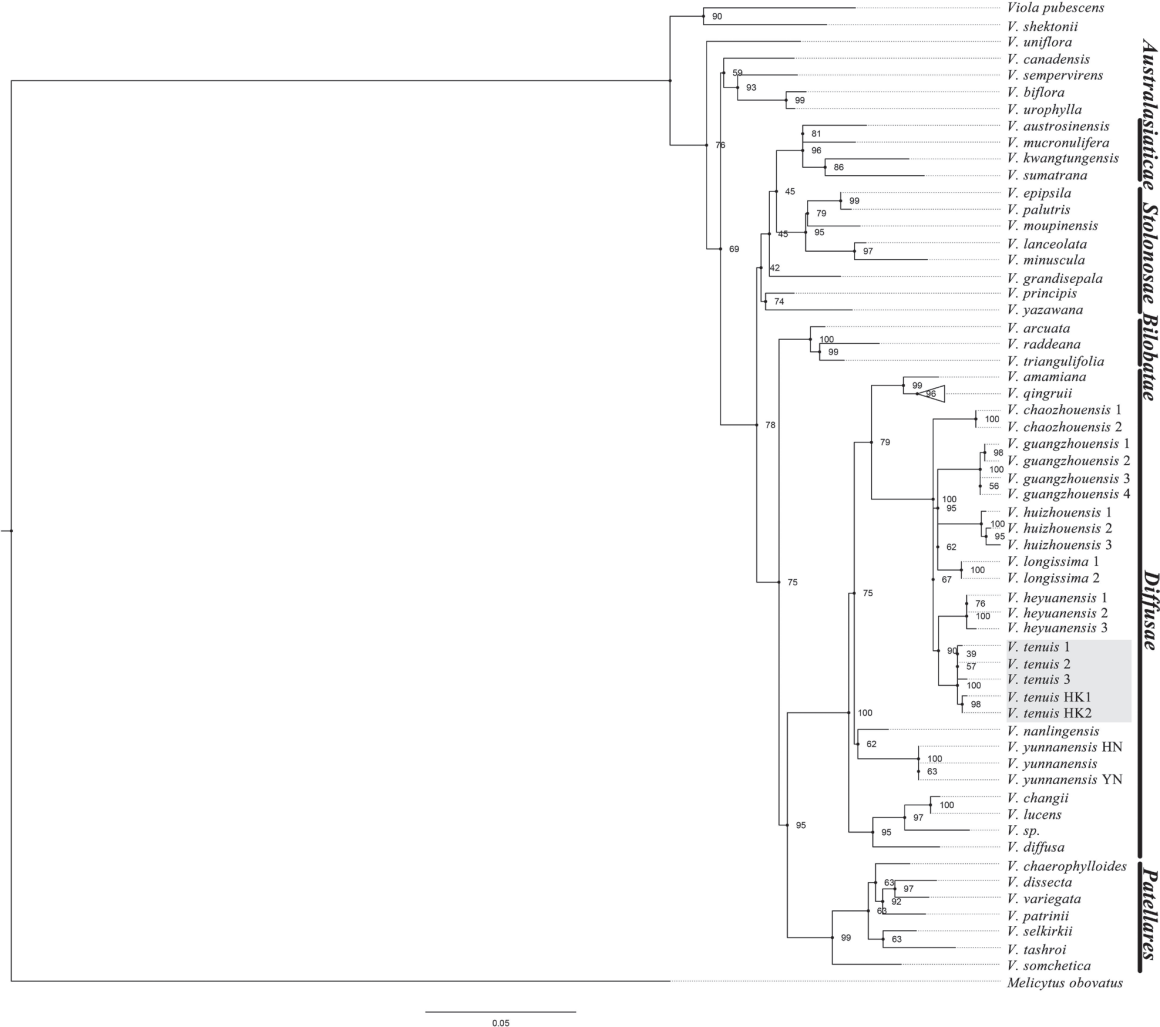
Maximum Likelihood (ML) tree of the new species and related species. Bootstrap values of the Maximum Likelihood are shown along the branches. Outgroups: *Melicytusobovatus*. *Violatenuis* are marked in grey.

The aligned length of our ITS sequences was 724 bps in total. *Violatenuis* belongs to the monophyletic group V.subsect.Diffusae (BS = 100%, Fig. 1) and specimens of *V.tenuis* from Shenzhen and Hong Kong form a sister group with *V.heyuanensis* Yan S.Huang, Q.L.Ye & Q.Fan (BS = 90%).

### ﻿Taxonomic treatment

#### ﻿ Viola tenuis

Taxon classificationPlantaeMalpighialesViolaceae

Benth. in London J. Bot. 1: 482 (1842)

409949DC-DFC1-538B-9B68-F30701DB3AB3

Fig. 2

≡ Violadiffusasubsp.tenuis W.Becker in Philipp. J. Sci. 19(6): 714 (1921).

##### Type.

China • Hong Kong, 1841, *Hinds s.n.* (holotype: K000370141 [photo!]).

##### Additional specimens examined.

China • Hong Kong, Sai Kung, 06 May 2016, *Danial Hang HKY0012* (KFBG, Fig. 3c); • Hong Kong, Sai Kung, 13 Mar 2018, *Jingang Liu JG0615* (KFBG, Fig. 3b); • Hong Kong, Sai Kung, 21 Jul 2023, *Jinlong Zhang JL1488* (KFBG); • Zhuhai, Dawanshan Island, 07 Nov 1973, *Yue73 73-3160* (IBSC); • Zhuhai, Dawanshan Island, 04 Nov 1973, *Yue73 73-3055* (IBSC); • Zhuhai, Dangan Island, 12 Jul 2017, *Ming Tan Tu TY3390* (CDBI); • Shenzhen, Qiniangshan Mountain, 18 Mar 2005, *Shouzhou Zhang & Liangqian Li 0259* (SZG); • Shenzhen, Qiniangshan Mountain, 17 May 2005, *Shouzhou Zhang & Liangqian Li 1528* (SZG); • Shenzhen, Qiniangshan Mountain, 16 Apr 2005, *Shouzhou Zhang & Liangqian Li 0196* (SZG); • Shenzhen, Qiniangshan Mountain, 16 Apr 2019, *Q. Fan 18910* (SYS);

**Figure 2.**
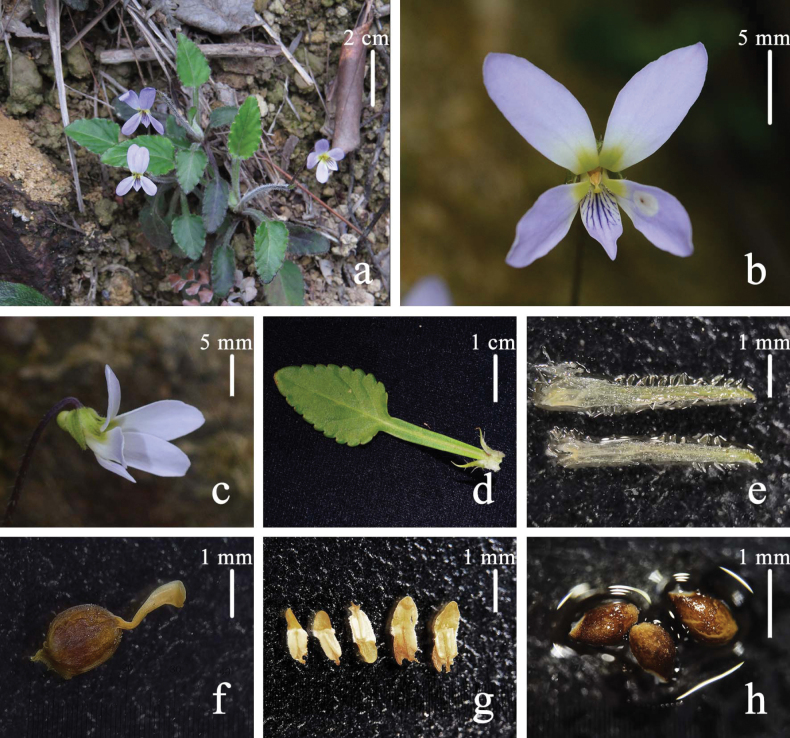
*Violatenuis* Benth. **a** habitat and habit (photograph in Hong Kong, J. Zhang) **b** flower, front view **c** flower, side view **d** leaf and stipules **e** bracteoles **f** ovary and stigma **g** stamens **h** seeds.

**Figure 3.**
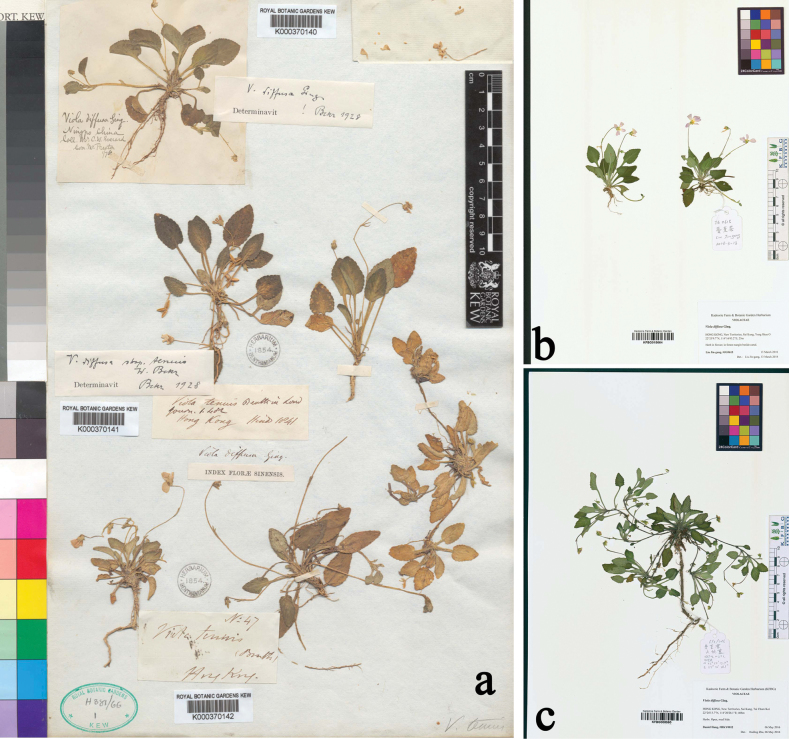
**a** holotype of *Violatenuis* Benth. (K000370141) **b, c** specimen of *V.tenuis* in KFBG (JG0615, HKY0012).

##### Etymology.

It is hypothesised that the specific epithet refers to the plant’s thin rhizomes.

##### Vernacular name.

We propose a Chinese name, Xì Jīng Jǐn Cài (细茎堇菜), to reﬂect the thin rhizomes of this species.

##### Description.

Perennial, stoloniferous herb with basal leaves rosulate, 5–8.5 cm tall. Rhizome erect or obliquely erect, rather slender, dark purple, with dense remains of stipules and petioles. Lateral stolons purple or light green, spreading and elongating, usually producing adventitious roots, with an apical rosette of leaves. Stipules ca. 8 mm, adnate to petioles for about 1/3 of their length at base, linear-lanceolate, acute at apex, with margins pinnatifid, glabrous or rarely puberulous. Petioles 2–4 cm, with wings narrow, glabrous or sparsely puberulous along the margin. Leaf blades ovate or ovate-oblong, 1.0–2.4 × 1.5–3.3 cm, glabrous or densely puberoulous; margin finely crenate; base wedge-shaped to truncated; apex obtuse, rarely acuminate. Chasmogamous flowers ca. 1.5 cm in diam.; peduncles slender, 6–7 cm long, glabrous, much higher above the basal leaves, with two opposite bracteoles above middle; bracteoles linear-lanceolate, ciliate, 5.5–6 mm long, apex acuminate or obtuse. Sepals green, ciliate, lanceolate, 1.5–1.7 × 4–5.5 mm, with entire margin, acuminate apex and truncate, with extremely short semicircular appendages. Petals purple, anterior one with apparent violet lines, posterior and lateral ones with a yellow to green patch at base; posterior petals, narrowly ovate to oblong, 4.5 × 12 mm, glabrous, with entire margin and obtuse apex; lateral petals with straight to slightly clavate hairs at the base, oblong, ca. 1.3 cm long, with entire margin, apex obtuse; anterior petal spatulate, 8 mm long, with a short, round spur, interior side of the spur puberulent. Stamens 5, unequal, puberulent; anther thecae ca. 0.9 mm long, with terminal appendages ca. 0.6–0.85 mm long; posterior appendages (nectar spurs) of the two anterior stamens ca. 0.95 mm long, triangular. Ovary ovoid, ca. 1.25 × 1.49 mm, glabrous; style clavate, ca. 1.35 mm long, conspicuously geniculate at base; stigma glabrous, with thickened lateral margins, shortly beaked at apex. Cleistogamous flowers ca. 3.2 mm long; peduncles ca. 6.5 mm long; bracteoles linear-lanceolate, puberulous, ca. 2.8 mm long, apex acute. Sepals green, glabrous or puberulous, lanceolate, ca. 0.8 × 3.2 mm, with entire margin, acuminate obtuse. Capsules glabrous, light green to yellow, ovoid to oblong, ca. 9 mm long. Seeds brown, oblongoid, ca. 1.4–1.55 mm long, with tubercles and inconspicuous elaiosomes.

##### Phenology.

Chasmogamous flowers from April to May, rarely October, cleistogamous flowers from May to September and fruits from April to October.

##### Distribution and habitat.

**Figure 4.**
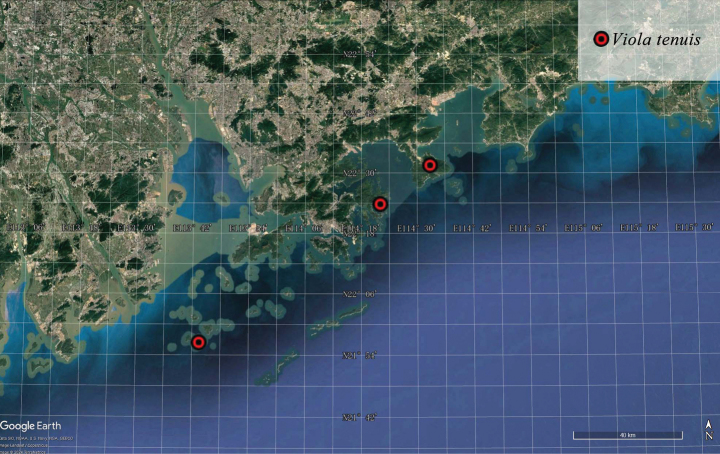
Distribution of *Violatenuis* Benth.

*Violatenuis* is currently known from Hong Kong, Shenzhen and Zhuhai in China (Fig. 4). The species grows on rocks or in rocky crevices at altitudes of 100–700 m a.s.l.

##### Conservation status.

As its area of occupancy is estimated less than 20 km^2^, the conservation status of *Violatenuis* should be considered as Vulnerable (VU) according to IUCN Red List criteria (D2; IUCN 2024).

## ﻿Discussion

Becker (1921), when merging *Violatenuis* into *V.diffusa* as a subspecies, described its leaves as roundish or ovate with a distinctly cordate base and long petioles. While accepting the subspecies, he suggested it might simply be a small-leaved variety of *V.diffusa*. However, Bentham (1842), when publishing this species, described it as having ovate-oblong leaves with a truncate base, along with rhizomes and stolons; he specifically mentioned that the leaves are similar to those of *V.patrinii* Ging. Although the original description did not include a type designation, there is only one specimen collected by Richard Brinsley Hinds in Hong Kong in 1841 (Fig. 3a). As the individuals on it also have narrow and truncate leaves, the distinct morphological characteristics clearly distinguish it from *V.diffusa* and other species of the *Diffusae* group. Based on our field surveys, *V.tenuis* Benth. is endemic to Guangdong and Hong Kong (Figs 3 and 4) and forms a paraphyletic group with *V.diffusa* (Fig. 1), so it is more appropriate to be treated as a separate species. Both the name *Violatenuis* proposed by Becker in 1907 and Viola×tenuis proposed by Karl Richter in 1891 are illegitimate later homonyms, based on Shenzhen Code Art. 11.4 and 53.1 (Turland et al. 2017).

In our phylogenetic tree, *V.tenuis*, *V.huizhouensis* Yan S.Huang & Q.Fan, *V.chaozhouensis* Yan S.Huang, J.H.Ding & Q.Fan, *V.guangzhouensis* A.Q.Dong, J.S.Zhou & F.W.Xing, *V.longissima* Yan S.Huang & Q.Fan and *V.heyuanensis* form a monophyletic group (Fig. 1). The latter three species have aerial stems, while the former three do not. The colour and hairiness of the leaf surface, the thickness of the rhizome and the size of the flowers can distinguish the first three species, but the relationship between morphological characters and the phylogeny still needs more investigation and evidence. These species are distributed in the mountainous area of Guangdong Province and appear to have undergone adaptive radiation (Huang et al. 2023). We hypothesise that sea level change might have influenced their diversification (Holbourn et al. 2018).

Based on our field surveys, molecular and morphological analyses (Table 1), we provide strong evidence supporting the recognition of *V.tenuis* Benth. as a distinct species and, thus, that this name should not be treated as a synonym.

**Table 1.** Morphological comparisons between *Violatenuis*, *V.diffusa* and *V.heyuanensis*.

Species name	*Violatenuis*	*Violadiffusa*	*Violaheyuanensis*
Leaf blade	Ovate to ovate-oblong, 1.0–2.4 × 1.5–3.3 cm, glabrous or puberulous; finely crenate margin; wedge-shaped to truncated base; obtuse apex, rarely acuminate	Ovate to oblong, 1.7–2.5 × 3.0–4.9 cm, nearly glabrous; crenate margin; wedge-shaped base, gradually tapering into petiole	Narrowly ovate to triangular, 1.8–3.2 × 1.3–1.8 cm, densely pubescent; coarsely serrate margin; cuneate to truncate base, rarely cordate; obtuse or acuminate apex
Petioles	2–4 × ca. 0.1–0.2 cm, glabrous or sparsely puberulent, narrow wings	1.6–2.0 × 0.35–0.47 cm, sparsely pubescent, broad wings	2–4.5 cm, densely pubescent, narrow wings
Aerial stem	Absent	Absent	Aerial stems solitary or several and fasciculate, erect, slender
Rhizome	Erect or obliquely erect, rather slender, dark purple, with dense remains of stipules and petioles	Absent, with fibrous root	Erect or obliquely erect, rather stout, reddish-brown rarely branched
Flower	Chasmogamous flowers ca. 1.5 cm in diam.	Chasmogamous flowers ca. 0.8 cm in diam.	Chasmogamous flowers ca 1.2 cm diam.
Seeds	Brown, oblongoid, with tubercles, 1.4–1.55 mm long	Yellow, obovoid, with smooth surface, 1–1.2 mm long	Brown, obovoid, with sparse tubercles, 1–1.5 mm long

There is no type specimen of *V.tenuis* Benth. designated in the protologue (Bentham 1842), which was entitled as ‘Enumeration of plants collected from Hong Kong, by RB Hinds, determined and described by George Bentham, ESQ’. According to the publication, we determined K000370142 (Fig. 2a) in the Royal Botanic Garden Kew as the holotype of *V.tenuis* (Shenzhen Code, Art. 7.9, Art. 8.1), as it is the only specimen collected by Hinds and identified as *Violatenuis* by G. Bentham.

### ﻿Key to Violasubsect.Diffusae

**Table:** 

1	Leaf base conspicuously decurrent to the petiole	**2**
–	Leaf base inconspicuously or not decurrent	**3**
2	Seeds with a conspicuous elaisome	***Violapendulipes***
–	Elaisome inconspicuous or absent	**4**
3	Leaf blades oblong to oblong–lanceolate, distinctly longer than broad	***V.yunnanensis***
–	Leaf blades ovate or orbicular, about as long as broad	**5**
4	Flowers 20–35 mm across	**6**
–	Flowers 4–20 mm across	**7**
5	Flowers with a yellow to green patch at the base	***V.qingruii***
–	Flowers without a yellow to green patch at the base	**8**
6	Plants with distinct aerial stems	**9**
–	Plants with short or no aerial stem	**10**
7	Leaf blades abaxially purple	***V.jinggangshanensis***
–	Leaf blades light green	**11**
8	Stipules triangular; flowers white	***V.lucens***
–	Stipules linear to linear-lanceolate; flowers light pink	***V.changii***
9	Leaf blades abaxially purple	***V.guangzhouensis***
–	Leaf blades light green	**12**
10	Leaves papery when dried	………………….…………… ***V.nanlingensis***
–	Leaves thinly leathery when dried	**13**
11	Seeds yellow to light brown, with smooth surface	**14**
–	Seeds dark brown, with tubercles	**15**
12	Posterior petals shorter than lateral ones; aerial stems 5–7 cm tall	***V.heyuanensis***
–	Posterior petals longer than lateral ones; aerial stems up to 40 cm tall	***V.longissima***
13	Leaves abaxially green; rhizomes slender	***V.tenuis***
–	Leaves abaxially dark purple	**16**
14	Lateral petals bearded	***V.diffusa***
–	Lateral petals glabrous	***V.amamiana***
15	Lateral petals bearded	***V.pricei***
–	Lateral petals glabrous	***V.nagasawae***
16	Leaf apex acute; leaf blade glabrous or pubescent only along veins	***V.chaozhouensis***
–	Leaf apex obtuse; leaf blade densely covered with white pubescence	***V.huizhouensis***

## Supplementary Material

XML Treatment for Viola tenuis

## ﻿Additional information

### Author ORCIDs

Jinlong Zhang https://orcid.org/0000-0002-1161-5460

Qiang Fan https://orcid.org/0000-0003-4254-6936

### Data availability

All of the data that support the findings of this study are available in the main text or Supplementary Information.

## Supplementary materials

Supplementary material 1 GenBank accessions for phylogenetic analysis Data type docx https://binary.pensoft.net/file/1256641This dataset is made available under the Open Database License (http://opendatacommons.org/licenses/odbl/1.0/). The Open Database License (ODbL) is a license agreement intended to allow users to freely share, modify, and use this Dataset while maintaining this same freedom for others, provided that the original source and author(s) are credited.Yan-Shuang Huang, Stephan W. Gale, Jinlong Zhang, Qiang Fan
