# Supplementary material for: ﻿On the identity and typification of Violatenuis Bentham (Violaceae)
Source: PhytoKeys. 2025 Feb 20;252:197–206. doi: 10.3897/phytokeys.252.138994 (PMC11868806; doi:10.3897/phytokeys.252.138994)
Supplement: Supplementary material 1 — GenBank accessions for phylogenetic analysis [file phytokeys-252-197_article-138994__-s001.docx]

Table S1. GenBank accessions for phylogenetic analysis

| *V. biflora* | DQ055348 |
| --- | --- |
| *V. canadensis* | AF097231,  MG234951 |
| *V. pubescens* | DQ006044 |
| *V. sempervirens* | MG235908 |
| *V. sheltonii* | AF097226,  AF097272 |
| *V. uniflora* | AY582167,  AY541600 |
| *V. urophylla* | MH117805 |
| *V. austrosinensis* | OM406228 |
| *V. kwangtungensis* | OM406230 |
| *V. mucronulifera* | FJ002910 |
| *V. sumatrana* | OM406231 |
| *V. hamiltoniana or V. verecunda* | AY928283 |
| *V. raddeana* | AY928279 |
| *V. triangulifolia* | FJ002912 |
| *V. amamiana* | JF830899 |
| *V. diffusa* | MH711723 |
| *V. guangzhouensis_*1 | MW683480 |
| *V. guangzhouensis_*2 | MW683479 |
| *V. guangzhouensis_*3 | *–* |
| *V. guangzhouensis_*4 | *–* |
| *V. huizhouensis_*1 | MW683486 |
| *V. huizhouensis_*2 | MW683485 |
| *V. huizhouensis_*3 | MW683484 |
| *V. lucens* | FJ002913 |
| *V. nanlingensis* | FJ002916 |
| *V. yunnanensis* | FJ002915 |
| *V. albida*  *V. chaerophylloides* | DQ787762 |
| *V. dissecta* | JQ950564 |
| *V. patrinii* | AY928298 |
| *V. selkirkii* | AY928307 |
| *V. somchetica* | HM851457 |
| *V. tashiroi* | JF830885 |
| *V. variegata* | KC330743 |
| *V. suecica or*  *V. epipsila* | MG237736 |
| *V. grandisepala* | FJ002903 |
| *V. lanceolata* | MG235616 |
| *V. minuscula or*  *V. macloskeyi*  *subsp. pallens* | AF097236,  AF097282 |
| *V. moupinensis* | FJ002900 |
| *V. palustris* | KX166144 |
| *V. principis* | FJ002904 |
| *V. yazawana* | AY928289 |
| *V. heyuanensis_*1 | OP935140 |
| *V. heyuanensis_*2 | OP935141 |
| *V. heyuanensis_*3 | OP935154 |
| *V. chaozhouensis_*1 | OP935142 |
| *V. chaozhouensis_*2 | OP935143 |
| *V. qingruii_*HY1 | OP935144 |
| *V. qingruii_*HY2 | OP935145 |
| *V. qingruii_*HY3 | OP935146 |
| *V. qingruii_*GZ1 | OP935150 |
| *V. qingruii_*GZ2 | OP935151 |
| *V. qingruii_*GZ3 | OP935152 |
| *V. qingruii_*GZ4 | OP935147 |
| *V. qingruii_*GZ5 | OP935148 |
| *V. qingruii_*GZ6 | OP935149 |
| *V. tenuis_*1 | OP935156 |
| *V. tenuis_*2 | OP935157 |
| *V. tenuis_*3 | OP935158 |
| *V. tenuis_*HK1 | PP083724 |
| *V. tenuis_*HK2 | PP083725 |
| *V. longissima_*1 | OP935160 |
| *V. longissima_*2 | OP935161 |
| *V. yunnanensis_*HN | OP935159 |
| *V. yunnanensis_*YN | PP083726 |
| *V. yunnanensis* | FJ002915 |
